# Corona-Krise: die transformative Rezession

**DOI:** 10.1007/s10273-020-2676-5

**Published:** 2020-07-06

**Authors:** Christian Hutter, Enzo Weber

**Affiliations:** 1 Inst. f. Arbeitsmarkt- und Berufsforschung, Regensburger Straße 104, 90478 Nürnberg, Deutschland; 2 Forschungsbereich A1, Inst. f. Arbeitsmarkt- und Berufsforschung, Regensburger Str. 100, 90478 Nürnberg, Deutschland

**Keywords:** J24, E24, O3

## Abstract

Mit der Corona-Krise nimmt die Arbeitslosigkeit trotz aller Gegenmaßnahmen in Deutschland zu. In früheren Rezessionen hat sich gerade die Arbeitslosigkeit Geringqualifizierter verfestigt. Heute zeichnet sich ab, dass der technologische Wandel gerade auch mittlere Qualifikationen betrifft. Um Verfestigung zu vermeiden, ist es wichtig, Neueinstellungen zu fördern, Qualifizierung zu unterstützen und berufliche Umorientierung zu ermöglichen.

## Abstract (en)

With the Corona crisis, unemployment is increasing in Germany despite all the countermeasures taken. In previous recessions, unemployment of the low-skilled in particular has become entrenched. Today, it is becoming apparent that technological change is also affecting medium-skilled workers in particular. In order to avoid consolidation, it is important to promote new hires, support qualification and enable vocational reorientation.

Innerhalb kürzester Zeit hat der Corona-Schock die deutsche Wirtschaft mit voller Wucht getroffen. Zwar wird mit immensem finanziellen Aufwand alles versucht, um die bestehenden Jobs und Betriebe zu retten. Auch verfügt Deutschland nach Jahren des Beschäftigungsaufschwungs über einen grundsätzlich sehr robusten Arbeitsmarkt. Aber angesichts der Dimension des wirtschaftlichen Schocks wäre es illusorisch zu glauben, die Krise ließe sich ohne eine deutliche Zunahme der Arbeitslosigkeit durchstehen. Wenn die Arbeitslosigkeit nach der Krise wieder abnimmt, wäre der Schock zwar schmerzhaft, aber heilbar. Nur: Das ist kein Selbstläufer.

## Verfestigung von Arbeitslosigkeit in Rezessionen

Auch in früheren Rezessionen war die Arbeitslosigkeit wegen des wegbrechenden Arbeitskräftebedarfs gestiegen. Es entstand also eine konjunkturelle Arbeitslosigkeit, die wieder verschwinden sollte, wenn die Konjunktur anzieht. Über Jahrzehnte hinweg war das in Deutschland aber nicht der Fall (vgl. Abbildung 1). Klinger und Weber (2016) zufolge haben sich im Laufe eines Jahres in Rezessionen fast zwei Drittel der konjunkturellen Arbeitslosigkeit in persistente, strukturelle Arbeitslosigkeit gewandelt. Das heißt, Arbeitslosigkeit, die einer vorübergehenden Konjunkturschwäche geschuldet war, hat sich über die Zeit verfestigt. Dafür sind verschiedene Gründe relevant. So kann Arbeitslosigkeit für Arbeitgeber einen negativen Signaleffekt bezüglich der Fähigkeiten und der Motivation des Arbeitnehmers haben. Je länger die Arbeitslosigkeit anhält, desto stärker kann sich auch tatsächlich Demotivation einstellen. Und über die Arbeitslosigkeitsphase hinweg können Arbeitserfahrung und Qualifikationen veralten. Letzteres spielte gerade während des Anstiegs der Arbeitslosigkeit seit den 1970er Jahren eine Rolle. Mit der Automatisierung klassischer Fabrikarbeit, der Computerisierung und der Etablierung des Internets hatten sich Anforderungen im Erwerbsleben deutlich gewandelt. Die Arbeitslosenquote der Niedrigqualifizierten stieg zeitweise auf mehr als 25 % (vgl. Abbildung 1). Fielen die Jobs in einer Rezession weg, kamen sie danach nicht einfach wieder. Somit treten die Arbeitsmarktwirkungen des technologisch-strukturellen Wandels ruckartig auf, obwohl der Wandel selbst durchaus kontinuierlich verlaufen kann.

## Qualifikationsverzerrter technologischer Wandel

Die dunkelblaue Linie in Abbildung 2 zeigt den Verlauf des qualifikationsverzerrten technologischen Wandels in Deutschland (Hutter und Weber, 2019). Dieser ist dadurch gekennzeichnet, dass er die Produktivität Hochqualifizierter im Vergleich zu Niedrigqualifizierten begünstigt. Automatisierung kann Technologien schaffen, die komplementär zu Hochqualifizierten sind und diesen zusätzliche Mittel an die Hand geben. Zudem können Innovationen substitutiv für Niedrigqualifizierte sein, sodass deren Jobs unter Druck geraten. Dadurch kommt es zu einem (qualifikatorischen) Mismatch im Arbeitsmarkt, der Anpassungsbedarf und Reibung erzeugt und im ungünstigen Fall Arbeitslosigkeit verfestigt. Über Jahrzehnte bis in die 2000er Jahre hinein trat ein starker, qualifikationsverzerrter technologischer Wandel auf. Entsprechend war die Arbeitslosenquote der Niedrigqualifizierten über einige Rezessionen weitaus stärker gestiegen als diejenige der Personen mit beruflichem oder akademischem Abschluss (vgl. Abbildung 1).

**Dr. Christian Hutter** ist wissenschaftlicher Mitarbeiter am Institut für Arbeitsmarkt- und Berufsforschung.

**Prof. Dr. Enzo Weber** ist dort Forschungsbereichsleiter und lehrt an der Universität Regensburg.

Abbildung 1
**Qualifikationsspezifische Arbeitslosenquoten**

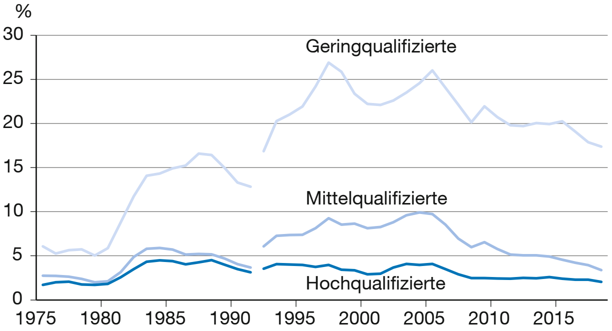

Anmerkungen: Geringqualifizierte: kein Berufsabschluss, Mittelqualifizierte: Lehre/Fachschule, Hochqualifizierte: Hoch-/Fachhochschule. Strukturbruch 1992 aufgrund der Wiedervereinigung.

Quelle: IAB-Berechnungen auf Basis Mikrozensus und Strukturerhebungen der Bundesagentur für Arbeit.

Anders war es hingegen in der Großen Rezession 2009, als es zu keiner Verfestigung kam. Dafür dürften verschiedene Gründe relevant sein. Klinger und Weber (2016) weisen auf die vorhergehenden Arbeitsmarktreformen hin, und Deutschland ging damals mit einem starken Aufwärtstrend am Arbeitsmarkt in die Krise (Weber, 2015). Dabei setzten sich günstige Trends wie die steigende Matchingeffizienz in der Krise fort (Hutter et al., 2019). Ein wichtiger Grund ist allerdings auch, dass in der Zeit der Weltfinanzkrise die technologischen Umbrüche, die niedrige Qualifikationen unter Druck setzen, deutlich schwächer ausgeprägt waren als in den Jahrzehnten zuvor (vgl. Abbildung 2, dunkelblaue Linie). Die Wellen der Computerisierung und des Internets waren bereits ausgelaufen, die von Industrie 4.0 und künstlicher Intelligenz aber noch nicht angelaufen (Hutter und Weber, 2019). Auch deshalb konnte wohl die Arbeitslosigkeit der Niedrigqualifizierten nach der Rezession zügig sogar unter das Vorkrisenniveau fallen.

## Technologischer Wandel: Qualifikationsebene steigt

Welche Situation ist heute und speziell für die Corona-Krise relevant? Die dunkelblaue Linie in Abbildung 2 zeigt, dass der qualifikationsverzerrte technologische Wandel bis zuletzt flach blieb. Einen derart starken Arbeitslosigkeitsabbau, wie er bis 2008 zu verzeichnen war, gab es trotz der relativ günstigen Entwicklung vor der aktuellen Corona-Krise aber nicht. Zudem ist der Arbeitsmarkt heute anders betroffen als in früheren Jahrzehnten. Das bisher betrachtete Maß des qualifikationsverzerrten technologischen Wandels bildet die Begünstigung von höheren und mittleren gegenüber niedrigeren Qualifikationen ab. Eine solche Begünstigung trat in den vergangenen Jahren nicht mehr auf. Wir haben nun aber zusätzlich dasselbe Maß für die Begünstigung hoher gegenüber niedrigen und mittleren Qualifikationen berechnet. Die hellblaue Linie in Abbildung

Abbildung 2
**Relative Faktorproduktivität nach Qualifikationen**

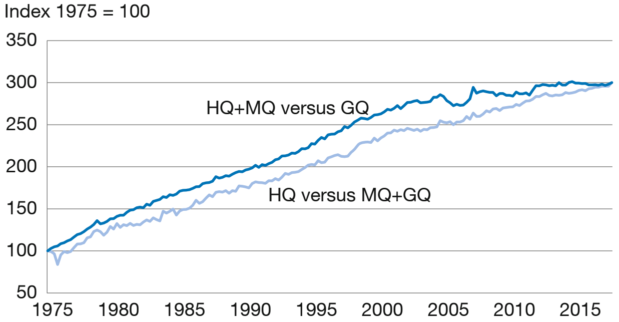

Anmerkungen: HQ + MQ versus GQ = Hochqualifizierte (Hoch-/Fachhochschulabschluss) und Mittelqualifizierte (abgeschlossene Berufsausbildung) abgegrenzt gegen Geringqualifizierte (GQ; kein Berufsabschluss). HQ versus MQ + GQ = Hochqualifizierte abgegrenzt gegen Mittel- und Geringqualifizierte.

Quelle: Stichprobe der Integrierten Arbeitsmarktbiografien (SIAB); eigene Berechnungen.

2 zeigt, dass diese zwar über die Jahrzehnte mit etwas geringerer Steigung voranschritt als das Maß ausschließlich zulasten der Niedrigqualifizierten, sich dafür aber zuletzt unverändert fortsetzte. Gerade berufliche Qualifikationen sind in den vergangenen Jahren also ins Hintertreffen geraten, der Wandel begünstigte die Hochqualifizierten.

Die heutige Situation kann also mit der Zeit der Verfestigung von Arbeitslosigkeit in Deutschland auf einer anderen Ebene durchaus vergleichbar sein: Die Entwicklung hin zu einer digitalen Wirtschaft 4.0 und die ökologische Transformation sind in vollem Gange. Die Jobs, die den deutschen Arbeitsmarktaufschwung ausmachten, können morgen ganz anders aussehen. In Wirtschaft-4.0-Szenarioanalysen wird ein deutlicher Trend zur Höherqualifizierung und starken Änderungen im mittleren Qualifikationsbereich erwartet (Wolter et al., 2016). Die Hauptrisiken liegen möglicherweise auf der mittleren Qualifikationsebene. Wenn hier Arbeitsplätze in der Rezession verschwinden, ist die Wahrscheinlichkeit hoch, dass sie nach der Rezession nicht wieder in derselben Form entstehen. Corona führt zu einer transformativen Rezession. Im Zentrum stehen trotz der aktuell akuten Betroffenheit dabei nicht unbedingt Einfacharbeitsplätze, sondern die Fachkraftebene – das Kernstück des deutschen Bildungssystems. Wir müssen das Risiko ernst nehmen, dass sich die in der Corona-Krise entstehende Arbeitslosigkeit verfestigen könnte. Derartige dauerhafte Schäden würden zu einer immensen sozialen und wirtschaftlichen Belastung führen.

## Wie kann Verfestigung vermieden werden?

In Deutschland werden normalerweise pro Jahr etliche Millionen sozialversicherungspflichtige Beschäftigungsverhältnisse neu begonnen – wenn diese ausbleiben, ist eine langwierige Arbeitslosigkeit vorprogrammiert. Genauso zeigt sich, dass die Arbeitsmarktintegration von Berufseinsteigern, die früh arbeitslos werden, dauerhaften Schaden nimmt (Möller und Umkehrer, 2015). Zudem drohen viele Menschen, die nach einer Entlassung bzw. dem Auslaufen einer Befristung normalerweise sofort den nächsten Job annehmen, jetzt in der Arbeitslosigkeit zu landen. Nach Ergebnissen der IAB-Stellenerhebung (Bossler et al., 2018) kam zuletzt rund die Hälfte aller Neueingestellten direkt aus anderen Jobs. Die Meldung offener Stellen hat sich gegenüber dem Vorkrisenniveau aber halbiert, und ausbleibende Neueinstellungen erhöhen die Arbeitslosigkeit mittlerweile stärker als beendete Beschäftigungsverhältnisse. Um Arbeitslosigkeit wieder abzubauen und Verfestigung zu vermeiden, schlagen Weber (2020a) und Merkl und Weber (2020) eine finanzielle Unterstützung von Neueinstellungen vor. Mit einem Rettungsschirm für Neueinstellungen würden bei den neuen Jobs über einen bestimmten Zeitraum die Sozialversicherungsbeiträge für Arbeitgeber und Arbeitnehmer ausgesetzt und stattdessen aus dem Bundeshaushalt finanziert. Der Rettungsschirm würde also als expansive Fiskalpolitik wirken, die nicht einfach nur breit streut, sondern gezielt Neueinstellungen unterstützt.

Zudem müssen gerade Zeiten der Rezession Zeiten der Weiterentwicklung und Qualifizierung sein. Um den Effekt des Veraltens von Qualifikationen zu vermeiden, wäre etwa ein Bildungsbonus geeignet, der laufend an alle gezahlt wird, die in der Arbeitslosigkeit eine geeignete längere Qualifizierung absolvieren. Dies würde Kompetenzen aktuell halten und eine nachhaltigere Entwicklung der Erwerbskarriere fördern. Wenn man etwa einen Betrag von 250 Euro pro Person und Monat annimmt, läge das finanzielle Volumen für ein Jahr bei bis zu 300 Mio. Euro. Insgesamt wird in der Weiterbildungspolitik ein proaktiverer Ansatz benötigt. Bestehende Regeln sehen etwa Fördermöglichkeiten für eine Person nur alle vier Jahre vor und das auch nur, wenn der Job vom Strukturwandel bedroht ist. So sehr die neuen Angebote zu begrüßen sind, zeigt sich dabei doch ein sehr defensiver, an Defiziten orientierter Ansatz: Es geht darum, Beschäftigte durch punktuelle Maßnahmen zu retten, wenn sie ins Hintertreffen geraten sind. Bei der Entscheidung für oder gegen Weiterbildung stellt sich aber gerade der Abstand vom Bildungssystem und vom Lernen an sich als wesentliche Hürde heraus. Um die Beteiligung entscheidend zu verbessern, wäre also eine substanzielle Förderung auf kontinuierlicher Basis nötig. Mehr Unterstützung für Weiterbildung käme gerade in der aktuellen Rezessionsphase genau richtig, in der viele Unternehmen unterausgelastete Kapazitäten, zugleich aber kaum Finanzmittel haben.

Schließlich wird die Transformation der Wirtschaft auch häufiger berufliche Umorientierung nötig machen. Neben dem Recht auf Nachholen eines Berufsabschlusses ist die zentrale Frage, unter welchen Bedingungen sich auch qualifizierte Beschäftigte auf den Weg zu einer neuen Ausbildung machen können. Dabei kann es um komplette Berufswechsel gehen, oder es wird innerhalb eines Berufsfelds die Ausrichtung geändert. Für derartige Entscheidungen kann mit professionellen Beratungsangeboten wichtige Orientierung gegeben werden. Dass Menschen mit Familie nach 20 Jahren im Job noch einmal für ein paar 100 Euro Ausbildungsvergütung in die Lehre gehen, darf aber als illusorisch gelten. Wir brauchen also für Zweitausbildungen ein BAFöG, das sich an den Bedarfen von Menschen in der Mitte des Berufslebens orientiert.
